# A386G polymorphism of the DAZL gene is not associated with idiopathic male infertility in North India

**DOI:** 10.4103/0974-1208.57222

**Published:** 2009

**Authors:** Kiran Singh, Rajiva Raman

**Affiliations:** Department of Molecular and Human Genetics and Zoology, Banaras Hindu University, Varanasi - 221 005, India; 1 Cytogenetics Laboratory, Banaras Hindu University, Varanasi - 221 005, India

**Keywords:** A386G polymorphism, azoospermia, *DAZL* gene, infertility, Y-chromosome microdeletion

## Abstract

**BACKGROUND:**

Male infertility is a multifactorial disorder which affects approximately 10% of couples at childbearing age with substantial clinical and social impact. Genetic variation and environmental factors contribute to susceptibility to spermatogenic impairment in humans. The A386G (T54A) polymorphism of the autosomal gene, *DAZL*, has shown susceptibility to spermatogenic failure in Taiwanese population. However, no such association has been seen in infertile patients from Italy and South India.

**AIM:**

This study aims to find out the possible association between A386G (T54A) polymorphism of the autosomal gene, *DAZL* and idiopathic male infertility in patients from North India.Case-control study.

**DESIGN:**

Case-control study.

**MATERIALS AND METHODS:**

The prevalence of A386G (T54A) polymorphism was determined in 165 idiopathic infertile azoo-/oligospermic patients and 200 fertile healthy control men. PCR-RFLP analysis was employed to determine the genotypes. PCR amplicons were subjected to restriction digestion with *Alu*I, as this mutation created a restriction site (AGCT), and separated on a 12% polyacrylamide gel.

**RESULTS:**

Analysis of 165 idiopathic infertile azoo-/oligospermic and 200 fertile control men revealed only one case of the variant as a heterozygote in the control population. Single Nucleotide Polymorphism (SNP) was absent in the infertile patients.

**CONCLUSION:**

As in the report from Italy and South India, our results illustrate the rarity of this mutation. Apparently, this mutation is of recent origin and/or has poor selective value. Its preponderance in infertile patients from Taiwan (all heterozygotes) suggests a founder effect and also that its low selective value could be due to impaired spermatogenesis.

## INTRODUCTION

In men, sex chromosomes and autosomes harbor genes which regulate spermatogenesis. Rearrangement or sequence variation in these genes is expected to lead to impaired spermatogenesis and reduced sperm count.[1,2] Nearly 10% idiopathic cases of nonobstructive azoo-/oligospermia are due to deletion of a specific region (AZF) on the long arm of Y-chromosome. The deleted region of Y comprises multiple copies of several genes; one of these, *DAZ* (Deleted in Azoospermia), is deleted in the vast majority of them. Association of single nucleotide polymorphisms (SNP) of genes with various multifactorial disorders is increasingly reported. We have already reported an association of C677T in MTHFR gene with idiopathic male infertility.[3] However, a report from Netherlands, for the same SNP, showed no association.[4] Apparently, the SNP mediated susceptibility to infertility may vary in a population specific manner.

*DAZL*, an autosomal gene (chromosome 3p24), has a high degree of homology with the Y-chromosomal *DAZ* whose deletion is one of the main genetic causes for azoo/oligospermia. Like its Y-chromosomal homologue, *DAZL* is expressed in germ cells encoding an RNA- binding protein.[5] It also has orthologues in other mammals, lower vertebrates and *Drosophila;* in all of them it is expressed in the germ cells. In mice, *DAZL*-knockouts are infertile due to loss of germ cells, and introduction of human *DAZ* partially rescues this loss of germ cells and fertility.[6] Unlike in mice, no infertility causing mutations have been identified in *DAZL* in man. Screening for *DAZL* mutants in a Taiwanese population has recorded an SNP at nucleotide 386 (A386G) in its cDNA which falls within the RNA recognition motif of the gene, and a threonine residue in the putative polypeptide chain would be replaced by alanine (T54A).[7] They show that the 386G allele does not express in the germ cell and the frequency of A386G heterozygotes in azoo-oligospermia probands is eight times higher than in fertile controls. Thus A386G in *DAZL* appears to be strong risk factor for azoo-/oligospermia.

There are, however, no other reported instances of a positive association of A386G polymorphism of the *DAZL* gene with idiopathic male infertility. There has been no study on infertile men from North India showing possible correlation between A386G polymorphism of the *DAZL* gene with idiopathic male infertility. To test the ubiquity of this in infertility, we have examined this SNP in a group of azoo-/oligospermic subjects and fertile men from a population in the north-eastern part of India to assess the phenotypic effect of this polymorphism.

## MATERIALS AND METHODS

A total of 165 infertile azoo-/oligospermic probands and 200 fertile controls of comparable age group (30, SD + 3), belonging to same geographical region, were recruited in the study. Informed consent was obtained from all the subjects to carry out molecular analyses and approval of institutional ethical committee was also obtained.

Patients married for a minimum of two years, having unprotected intercourse were considered for the present study. Three seminal fluid examinations were carried out after three/four days of sexual abstinence to ascertain their infertility status. The size, volume and consistency of testis, occurrence of varicocele, hydrocele or absence of secondary sexual characters were also recorded. Each patient's family history, habits (smoking, alcoholism etc.) and disease were recorded through a questionnaire. Those who suffered from varicocele, diabetes, mumps, obstruction, orchitis etc. were excluded from the study.

DNA was extracted from all the subjects and PCR from exon3 mutation was done using the previously prescribed primers.[7] PCR amplicons were subjected to restriction digestion with *Alu* I, as this mutation created a restriction site (AGCT), and separated on a 12% polyacrylamide gel. In the wild type *Alu* I digested DNA would yield two fragments of 115 and 66 bp while in the mutant another cut occurred within the 66bp fragment showing three bands: 115, 53 and 13 bp. In heterozygotes, three bands, 115, 66 and 53 bp bands were encountered.

## RESULTS AND DISCUSSION

**Figure 1:**
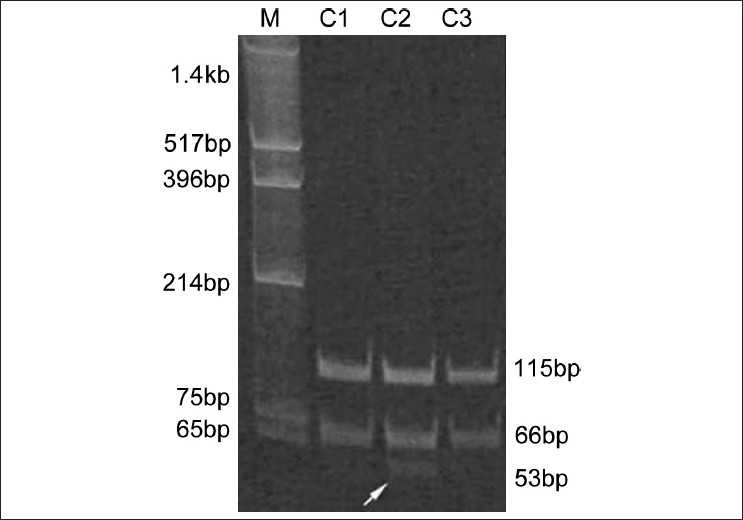
PCR-RFLP in controls for A386G mutation in *DAZL*. The 181bp PCR product was digested with Alu 1 and resolved on 12% polyacrylamide gel. Lane1 shows the marker (pUC/Hinf1), lane C1: AA homozygous, lane C2: AG heterozygous, lane C3: AA homozygous

A total of 365 individuals (165 infertile patients and 200 fertile controls) were examined. None among the infertile patients showed the SNP. Among the fertile controls, one individual was heterozygous for the SNP [Figure 1].

In addition to the present report, those by Teng *et al*.[7] and Bartoloni *et al*.[8] from Italy, Poongothai *et al.*[9] and Thangraj *et al.*,[10] from South India have analyzed the frequency of this SNP. All the three studies did not find the A386G polymorphism in the *DAZL* gene. The Bartoloni *et al*.[8] suggest that this mutation is nonexistent in the Caucasian population and must be of a recent origin in Asia. Therefore, they concluded, *DAZL* may have no role to play in infertility in European populations. In fact, excluding the infertile cases reported by Teng *et al*. the overall frequency of G allele (leading to A phenotype) is indeed very low <0.002 (0/316 alleles in Italian, one in 730 in Indian, two in 464 in Taiwanese normal; total three in 1510). The relative rarity of this SNP supports its recent origin in Europe as well as Asian populations. In addition, absence of homozygotes, even in Taiwanese infertile patients, points to its low selective value apart from recent origin.

We believe that low selective value of the SNP may in fact be due to impaired spermatogenesis leading to infertility as seen in the Taiwanese population. However, it is also likely that A → G induced impairment could at least partly be rescued by the Y-chromosomal *DAZ*. It is noteworthy that human Y-chromosomal *DAZ* is an evolutionary novelty occurring only in select group of primates. That is, a *DAZL* mutation in any other species would have a more severe phenotype than in man. We consider that one of the reasons for fertility of three men with this mutation; infertile in four of the A → G Taiwanese infertile patients also have *DAZ* deletion.

It is curious that when this SNP is so low in such diverse populations as Indian (Asian) and Italian (Caucasian) global, it should occur in such high proportion in Taiwan, more specifically in the infertile patients. Teng *et al*.[7] studied that the cases are representative of the whole Taiwanese population including all the different ethnic groups. It brings into consideration the role of the local environment and haplotypes of the Taiwanese population which make them more susceptible to this mutation. It would be of particular interest to analyze the haplotypes of the multicopy *DAZ* in this population.
